# A context-dependent METTL1-m7G-SLC7A11 axis links metabolic stress to epithelial fate in ulcerative colitis

**DOI:** 10.7150/ijbs.133562

**Published:** 2026-05-11

**Authors:** Lichao Yang, Qi Sun, Zhixian Jiang, Baojia Yao, Caimei Yang, Ganglei Liu, Lianwen Yuan

**Affiliations:** 1 Department of General Surgery, The Second Xiangya Hospital of Central South University, 410011 Changsha, China.; 2 Center for Clinical Sciences, Japan Institute for Health Security, Tokyo, Japan.

**Keywords:** Ulcerative colitis, SLC7A11, METTL1, N7-methylguanosine (m7G), Disulfidptosis, Metabolic stress.

## Abstract

Ulcerative colitis (UC) is characterized by chronic intestinal inflammation accompanied by epithelial barrier dysfunction and profound metabolic stress; however, how metabolic cues are integrated to determine epithelial cell fate remains incompletely understood. Here, we identify a context-dependent METTL1-m7G-SLC7A11 regulatory axis that links metabolic stress to intestinal epithelial outcomes during UC progression. By integrating analyses of human UC tissues, DSS-induced acute and chronic colitis mouse models, and mechanistic *in vitro* experiments, we demonstrate that METTL1 enhances N7-methylguanosine (m7G) modification of SLC7A11 mRNA, thereby stabilizing the transcript and sustaining SLC7A11 expression in inflammatory settings. Functionally, SLC7A11 exhibits glucose-dependent dual effects. Under glucose-replete conditions, SLC7A11 supports cystine uptake, glutathione synthesis, and redox homeostasis, protecting epithelial integrity and limiting inflammation. In contrast, under glucose deprivation—a characteristic feature of inflamed UC mucosa—persistent SLC7A11 activation induces disulfide stress, cytoskeletal collapse, and disulfidptosis-associated epithelial injury. *In vivo*, inhibition of the METTL1/m7G/SLC7A11 axis exacerbates chronic DSS-induced colitis but alleviates acute DSS-induced colitis, revealing a switch from adaptive to maladaptive signaling with escalating metabolic stress. Collectively, these findings establish the METTL1-m7G-SLC7A11 axis as a metabolic rheostat that integrates inflammatory cues and nutrient availability to determine epithelial cell fate in UC, highlighting the importance of stage- and context-specific therapeutic strategies.

## Introduction

Ulcerative colitis (UC) is a type of inflammatory bowel disease (IBD), and its clinical features include persistent superficial mucosal ulceration, colorectal bleeding, diarrhea, and abdominal pain 1. The lesions of UC are confined to the colorectum, and inflammatory infiltration is mainly limited to the mucosal layer. Its pathogenesis is considered to be multifactorial, involving genetic susceptibility, environmental factors, lifestyle, intestinal immune imbalance, and gut microbiota dysbiosis 2. In recent years, the global incidence of UC has shown a continuous upward trend, posing a serious threat to both physical and mental health and imposing a substantial economic burden on patients and society 2-4. To date, the clinical management of UC primarily relies on pharmacological therapies, including aminosalicylates, corticosteroids, immunosuppressants, and biological agents 1,5. However, individual responses to these treatments vary considerably, and long-term and repeated drug administration is associated with notable limitations, such as significant side effects, poor drug tolerance, and limited long-term maintenance efficacy 4,6.

Although the precise pathogenesis of UC has not yet been fully elucidated, dysfunction of the intestinal mucosal barrier is considered one of the key initiating factors, and abnormal death of intestinal epithelial cells can directly lead to damage of this barrier 7. Among the various forms of cell death, a newly identified, metabolic stress-dependent form known as disulfidptosis has recently been demonstrated by our study to be significantly associated with disease severity and biological therapy response in UC 8. As a chronic inflammatory disease, UC is characterized by persistently elevated metabolic demands in the affected tissues. Continuous glucose consumption and impaired glucose absorption in the local microenvironment may expose epithelial cells to glucose-deprived metabolic stress, a condition that has been shown to be one of the critical prerequisites for the induction of disulfidptosis 9,10. Multiple studies have reported the anti-inflammatory roles of SLC7A11 (xCT), a key driver of disulfidptosis 11-13. Conversely, other studies have shown that high expression of SLC7A11 under conditions of metabolic dysregulation, such as glucose deprivation, can induce disulfide stress in tumor cells, leading to cytoskeletal collapse 14,15. Although no studies have yet directly demonstrated the presence of disulfidptosis in UC, this context-dependent functional switch suggests that SLC7A11 may exert dual roles during the dynamic inflammatory progression of UC.

In addition, our previous studies revealed that N7-methylguanosine (m7G) RNA methylation is closely associated with disease progression and biological therapy response in UC, suggesting that epitranscriptomic remodeling represents an important regulatory layer linking the inflammatory microenvironment to changes in cellular phenotypes 16. The m7G modification mediated by methyltransferase-like 1 (METTL1) has been demonstrated to occur on mRNA and to significantly influence mRNA stability, splicing, and translational efficiency, and it is closely correlated with the expression of inflammation-related signaling pathways 17. However, current functional studies on METTL1 have mainly focused on the field of cancer, and its biological significance in chronic inflammatory diseases remains largely unexplored 18,19, particularly with regard to whether and how METTL1-mediated m7G modification participates in the regulation of intestinal epithelial cell fate.

Therefore, based on the combined roles of m7G modification and disulfidptosis in UC-associated intestinal inflammation, we hypothesize that during the early stage of UC or under mild inflammatory conditions, activation of the “METTL1-m7G-SLC7A11” regulatory axis enhances SLC7A11-mediated antioxidant defense, thereby helping epithelial cells to resist stress and maintain barrier integrity, which manifests as a protective compensatory response. However, under sustained and severe inflammatory and metabolic stress, persistent activation of this axis may lead to continuous overexpression of SLC7A11, thereby triggering disulfidptosis, exacerbating epithelial injury, and promoting a vicious cycle of inflammation, ultimately entering a pathological decompensated state.

## Materials and Methods

### Human UC intestinal tissue specimens

Intestinal tissue specimens from patients with UC were obtained from individuals who underwent total colectomy with partial rectal resection at the Department of General Geriatric Surgery, The Second Xiangya Hospital of Central South University. The diagnosis of UC and the inclusion/exclusion criteria were determined according to the diagnostic guidelines issued by the European Crohn's and Colitis Organisation (ECCO) 20. Relatively normal colonic tissues were collected from macroscopically unaffected regions of the same surgical specimens and were further confirmed to be histologically normal by postoperative pathological examination. Written informed consent was obtained from all patients prior to sample collection. Fresh tissue samples were immediately snap-frozen in liquid nitrogen and subsequently stored at -80 °C for long-term preservation until further use. The collection and use of human tissue samples were approved by the Medical Ethics Committee of The Second Xiangya Hospital of Central South University (approval number: 2020580) and were conducted in accordance with the principles of the Declaration of Helsinki.

### GEO datasets

We systematically screened transcriptomic datasets derived from colonic mucosal tissues of patients with ulcerative colitis in the Gene Expression Omnibus (GEO) database to evaluate the expression patterns of SLC7A11. The included GEO datasets were GSE206171, GSE87466, GSE59071, and GSE107499. Specifically, GSE206171 comprised 114 UC samples and 38 healthy controls; GSE87466 included 87 UC samples and 21 controls; GSE59071 consisted of 74 UC samples and 11 controls; and GSE107499 contained 75 UC samples and 44 controls. In addition, to explore the potential association between METTL1-mediated m7G modification and SLC7A11 transcripts, we retrieved m7G MeRIP-seq data of mammalian mRNAs from the GSE112276 dataset, which includes sequencing data from HEK293T and HeLa cells 21. Datasets were selected based on sample size, tissue relevance (colon mucosa), and the availability of raw or normalized expression data.

### RNA sequencing and data analysis

To explore transcriptomic alterations induced by METTL1 knockdown in FHC cells, RNA sequencing was performed on sh-METTL1 and sh-Ctrl groups. Total RNA was extracted from FHC cells using TRIzol reagent according to the manufacturer's instructions. RNA concentration and integrity were assessed prior to library preparation. RNA-seq libraries were constructed using the Illumina TruSeq RNA Library Preparation Kit following the standard protocol. Briefly, poly(A)+ RNA was enriched from total RNA, fragmented, and reverse-transcribed to generate cDNA libraries, which were subsequently indexed and amplified for paired-end sequencing. Sequencing was conducted on the Illumina HiSeq 2500 platform (platform ID: GPL16791). All raw sequencing data have been deposited in the GEO database under the accession number GSE315761. For transcriptomic analysis, differential expression was assessed using the limma, DESeq2, and edgeR packages. Genes with an absolute log2 fold change (|log2FC|) ≥ 1 and a P value < 0.05 were considered differentially expressed. False discovery rate (FDR) correction was applied where applicable. The selected differentially expressed genes were subsequently subjected to Kyoto Encyclopedia of Genes and Genomes (KEGG) pathway enrichment analysis and Gene Ontology (GO) functional annotation. All R scripts used for transcriptomic data processing and analysis are provided in the Supplementary Materials.

### Cell culture and treatments

Normal human colonic epithelial cells (FHC and NCM460) were obtained from the Cell Bank of the Chinese Academy of Sciences (Shanghai, China; GNHu74) and from Otwobiotech (China; HTX1841C). Cells were maintained in DMEM (Gibco) supplemented with 10% fetal bovine serum (FBS; ExCell, FSD500) and 1% penicillin-streptomycin (P/S; NCM Biotech, C100C5) at 37 °C in a humidified incubator with 5% CO₂. To establish an *in vitro* inflammatory model, cells were stimulated with lipopolysaccharide (LPS; Sigma-Aldrich, L2880) at a final concentration of 50 ng/mL. To mimic metabolically constrained conditions, culture medium was replaced with glucose-free DMEM (Thermo Fisher Scientific, 11966025) as indicated. Cells were harvested at the specified time points for subsequent analyses.

### siRNA

Small interfering RNAs (siRNAs) were designed and synthesized by Shanghai Genechem. The detailed siRNA sequences are provided in the Supplementary Materials. Transfections were performed using Lipo8000™ transfection reagent (Beyotime Biotechnology, C0533) according to the manufacturer's instructions. A non-targeting siRNA was used as a negative control. Knockdown efficiency was validated by qPCR and/or western blotting.

### Plasmid construction and lentiviral transduction

Lentiviral-mediated knockdown and overexpression of METTL1 and SLC7A11 were performed in cultured cells. All lentiviral constructs were designed, generated, and packaged by Shanghai GeneChem, and detailed sequence information is provided in the Supplementary Materials. After transduction, cells were selected using puromycin at a concentration of 10 μg/mL and subsequently maintained in medium containing 2 μg/mL puromycin. The efficiency of gene knockdown or overexpression was validated by qPCR and/or western blotting.

### AAV vector construction and *in vivo* transduction

Adenoviral vectors targeting METTL1 and SLC7A11 were designed, constructed, packaged, and purified by Shanghai GeneChem. All vectors were knockdown constructs, and detailed targeting sequences are provided in the Supplementary Materials. For METTL1 (mouse gene ID: 17299, transcript: NM_010792.1) and SLC7A11 (mouse gene ID: 26570, transcript: NM_011990.2), an intestinal epithelial cell-specific promoter vector (CV762: villin promoter-EGFP-MIR155(mcs)-TK PolyA) was used. All vectors carried EGFP as a reporter and were based on the AAV9 serotype, with a viral titer of 1 × 10¹³ VG/mL. Given the epithelial focus of this study, adenoviral delivery was achieved via intrarectal administration. Mice were euthanized approximately 2-3 weeks after viral administration, and knockdown efficiency was validated by qPCR and/or western blotting.

### Cell Counting Kit-8 (CCK8)

Cell viability was assessed using a Cell Counting Kit-8 assay (Beyotime Biotechnology, C0037). Cells were seeded at equal densities in 96-well plates and subjected to the indicated treatments. CCK-8 reagent was added and incubated according to the manufacturer's instructions, and absorbance was measured using a microplate reader. Data were expressed as relative cell viability normalized to control.

### Flow cytometry

Apoptosis was assessed using an Annexin V/propidium iodide (PI) double-staining assay with a commercial kit (Jiangsu Keygen Biotech, KGA1030). After the indicated treatments, cells were collected, stained according to the manufacturer's instructions, and analyzed using a flow cytometer (Beckman, A00-1-1102). Debris was excluded based on forward scatter (FSC) and side scatter (SSC), and singlets were gated. Quadrant thresholds were defined using unstained and single-stained controls and were kept consistent within each acquisition batch. The proportion of PI-positive cells was used to estimate the extent of cell death. Data were analyzed using CytExpert software.

### Morphological assessment of disulfidptosis

To assess morphological features associated with disulfidptosis, cytoskeletal organization was visualized by Phalloidin-F-actin staining. These experiments were performed by Wuhan Servicebio Technology using a commercial Phalloidin-F-actin staining kit (Servicebio, GDP1021). Fluorescence images were acquired using an ultra-high-resolution confocal microscope (Leica, STELLARIS 5). Representative images were used to illustrate cytoskeletal alterations.

### Measurement of metabolic indicators

Intracellular redox homeostasis and energy metabolism were assessed by measuring reduced and oxidized glutathione (GSH/GSSG), nicotinamide adenine dinucleotide phosphate (NADP⁺/NADPH), and adenosine triphosphate (ATP) levels in both cells and colon tissues. Commercially available assay kits were used for all measurements, including GSH/GSSG (Beyotime Biotechnology, S0053), NADP⁺/NADPH (Beyotime Biotechnology, S0179), and ATP (Beyotime Biotechnology, S0027). All assays were performed according to the manufacturers' instructions. Data were normalized as appropriate based on the experimental design.

### RT-qPCR

Total RNA was extracted from cells or tissues using a commercial RNA isolation kit (Accurate Biology, AG21017) and reverse-transcribed into cDNA using a reverse transcription kit (ABCLONAL, RK20429). Quantitative PCR was performed using a SYBR Green-based system on a real-time PCR instrument. Relative gene expression levels were calculated using the 2^-ΔΔCt method and normalized to housekeeping genes. Primer sequences are provided in the Supplementary Table.

### Western blotting

Cells or colon tissues were lysed in RIPA buffer supplemented with protease and phosphatase inhibitors. Protein concentrations were determined using the BCA assay, and equal amounts of protein were loaded for each sample. Proteins were separated by SDS-PAGE and transferred onto PVDF membranes, with a prestained protein ladder (NCM Biotech, P9006) used as the molecular weight marker. After blocking, membranes were incubated with appropriate primary antibodies followed by HRP-conjugated secondary antibodies. Signals were visualized by chemiluminescence and quantified using ImageJ or equivalent software. Target protein levels were normalized to loading controls. Detailed information on all antibodies, including suppliers, catalog numbers, and dilutions, is provided in the Supplementary Materials. GAPDH or α-tubulin was used as an internal loading control for Western blot analysis. Considering that glucose deprivation-induced metabolic stress may affect GAPDH expression, α-tubulin was used as an additional control in selected experiments to ensure the stability and reliability of normalization.

### m7G dot blotting

To assess global m7G modification levels in total RNA, a dot blot assay was performed. Briefly, RNA samples extracted from cells or tissues were thawed on ice, quantified, adjusted to equal concentrations, and serially diluted. RNA samples were then denatured at 95 °C for 3 min to disrupt secondary structures and immediately cooled on ice. Equal volumes of RNA were spotted onto nylon membranes (Solarbio, YA1760) and allowed to air-dry. RNA was subsequently immobilized onto the membrane by UV crosslinking at 254 nm. After washing with TBST and blocking, membranes were incubated with an m7G-specific primary antibody at 4°C overnight, followed by incubation with HRP-conjugated secondary antibodies. Signals were visualized using enhanced chemiluminescence (ECL). To verify equal RNA loading, membranes were subsequently stained with methylene blue and imaged under white light as a loading control. Detailed information on all antibodies is provided in the Supplementary Materials. Dot blot was used as a semi-quantitative approach to evaluate global m7G levels.

### m7G MeRIP qPCR

To assess the m7G modification levels of SLC7A11 transcripts, m7G RNA immunoprecipitation followed by quantitative PCR (m7G MeRIP-qPCR) was performed. A commercial m7G MeRIP kit was purchased from Bersinbio (Bes5205), and all procedures were conducted according to the manufacturer's instructions. Briefly, total RNA was extracted from cells, fragmented, and subjected to immunoprecipitation using an antibody specific for m7G-modified RNA. The enriched RNA was then purified, reverse-transcribed, and quantified by qPCR. MeRIP signals were expressed as fold enrichment of immunoprecipitated RNA (IP) relative to input RNA, and further compared among different experimental groups. Primer sequences used for qPCR are provided in the Supplementary Materials.

For the detection of m7G-modified SLC7A11 transcripts, primer pairs were designed based on publicly available m7G-seq data downloaded from the m7GHub database 22. Three pairs of primers targeting the coding sequence (CDS) region of SLC7A11 were designed to cover the predicted m7G-modified sites. The exact primer sequences are provided in the Supplementary Materials.

### Enzyme-linked immunosorbent assay (ELISA)

Cytokine levels in cell culture supernatants or tissue homogenates were measured using commercially available ELISA kits. Detailed information on all ELISA kits, including suppliers and catalog numbers, is provided in the Supplementary Materials. In the LPS-induced epithelial cell model, IL-1β and IL-18 remained at consistently low or near-detection-limit levels; therefore, only TNF-α and IL-6 were quantified in the cell culture supernatants. All assays were performed according to the manufacturers' instructions, and cytokine concentrations were calculated based on standard curves.

### Chronic DSS-induced colitis mouse model

A chronic colitis mouse model was established using dextran sulfate sodium (DSS). DSS was purchased from MeilunBio (MB5535-2) and administered at a concentration of 1-2% (w/v). Female C57BL/6J mice (6 weeks old, SPF grade) were obtained from Hunan Silek Jingda Experimental Animal Co., Ltd. All mice were housed under specific pathogen-free (SPF) conditions at the Department of Laboratory Animals of Central South University, with *ad libitum* access to food and water. Mice were randomly assigned into six experimental groups (n = 6 per group): 1) Control; 2) DSS; 3) DSS+ sh-Ctrl; 4) DSS+ shMETTL1; 5) DSS+ shSLC7A1; and 6) DSS+ TCEP.

To induce chronic colitis, mice in the DSS-treated groups were subjected to three cycles of DSS administration. Each cycle consisted of 7 days of 1-2% (w/v) DSS in drinking water, followed by 14 days of regular drinking water for recovery. Body weight was recorded weekly throughout the experiment, and disease activity index (DAI) scores were assessed to monitor disease progression. Detailed criteria for DAI scoring were adopted from our previously published study 23.

At the end of the second recovery period (week 6), mice in the AAV-treated groups received a single intrarectal administration of the corresponding AAV vectors (1 × 10¹¹ viral genomes in 100 µL of PBS) under mild anesthesia, followed by the third and final DSS cycle. Mice were euthanized at the end of the third DSS cycle (week 9). The entire colon was rapidly excised from the cecum to the anus. Colon length was measured as a macroscopic indicator of disease severity. A segment of the distal colon was fixed in 4% paraformaldehyde for histology, while the remaining tissue was snap-frozen in liquid nitrogen and stored at -80 °C for subsequent molecular and biochemical analyses. All animal procedures were approved by the Animal Ethics Committee of Central South University (approval number: CSU-2023-0352) and conducted in accordance with the institutional guidelines for the care and use of laboratory animals.

### Acute DSS-induced colitis mouse model

An acute DSS-induced colitis model was established to mimic the decompensated inflammatory state of the intestinal epithelium and to investigate the effects of METTL1 and SLC7A11 on disulfidptosis under severe metabolic stress conditions. Mice were administered 4-5% (w/v) DSS in drinking water continuously for 7 days to induce acute colitis.

Body weight and disease activity index (DAI) scores were monitored daily throughout the modeling period. Mice were euthanized on day 7, and the entire colon was excised from the cecum to the anus. Colon length was measured as a macroscopic indicator of disease severity. For mice receiving AAV-mediated gene knockdown, intrarectal administration of the corresponding AAV vectors was performed 2 weeks prior to DSS treatment to ensure optimal gene silencing efficiency before colitis induction. Mice were randomly assigned into the following five groups (n = 6 per group): DSS; DSS + sh-Ctrl; DSS + sh-METTL1; DSS + sh-SLC7A11; DSS + TCEP. This acute DSS model was specifically used to evaluate the effects of METTL1 and SLC7A11 on epithelial fate and disulfidptosis under conditions of severe inflammation and metabolic dysregulation.

### Hematoxylin and eosin (H&E) staining

Freshly harvested colon tissues were rinsed with PBS to remove residual contents and immediately fixed in a commercial tissue fixative (Servicebio, G1101). Samples were subsequently processed for paraffin embedding by Wuhan Servicebio Technology. Paraffin sections were deparaffinized, rehydrated, and subjected to standard hematoxylin and eosin (H&E) staining. Stained sections were examined under a light microscope, and representative images were acquired.

### Immunohistochemistry (IHC)

Paraffin-embedded colon sections were deparaffinized and rehydrated, followed by antigen retrieval to restore antigenicity. Endogenous peroxidase activity was quenched using 3% hydrogen peroxide, and nonspecific binding was blocked with normal serum. Sections were then incubated overnight at 4 °C with primary antibodies against METTL1, SLC7A11, and m7G. After incubation with HRP-conjugated secondary antibodies, signals were visualized using a diaminobenzidine (DAB) substrate system. Nuclei were counterstained with hematoxylin. Stained sections were examined under a light microscope, and representative images were acquired. Detailed information on all antibodies, including suppliers, catalog numbers, and dilutions, is provided in the Supplementary Materials.

### Immunofluorescence (IF)

For tissue samples, paraffin-embedded colon sections were deparaffinized, rehydrated, and subjected to antigen retrieval. After blocking with normal serum, sections were incubated overnight at 4 °C with primary antibodies against METTL1 or SLC7A11. Sections were then incubated with appropriate fluorescently labeled secondary antibodies, and nuclei were counterstained with DAPI. For cell samples, F-actin organization was visualized by Phalloidin staining to assess cytoskeletal alterations associated with disulfidptosis. After staining, images were acquired using a confocal microscope. All fluorescence images were captured using a confocal microscope (Leica STELLARIS 5). Detailed information on all antibodies, including suppliers, catalog numbers, and dilutions, is provided in the Supplementary Materials. To objectively quantify immunofluorescence signals, image analysis was performed using ImageJ software. Briefly, images were converted to 8-bit grayscale. A consistent threshold was applied across all images within an experimental set to differentiate specific positive signals from background. For each field of view, either the Mean Fluorescence Intensity (MFI) or the percentage of positive area was measured. A minimum of three random fields per sample were analyzed, and results were compared as group means.

### Statistical analysis

All statistical analyses and data visualization were performed using GraphPad Prism 9.3.0 and R software (version 4.5.1). Unless otherwise specified, all *in vitro* biological experiments were independently repeated at least three times. Data are presented as mean ± standard deviation (SD) or mean ± standard error of the mean (SEM), as indicated in the figure legends. Comparisons between two groups were performed using two-tailed Student's t-tests, while comparisons among multiple groups were conducted using one-way or two-way analysis of variance (ANOVA), followed by appropriate post hoc tests when applicable. A P value < 0.05 was considered statistically significant. 'n' represents the number of biologically independent samples or animals.

## Results

### Metabolic dysregulation and glucose deprivation are prevalent in the UC intestine and accompanied by elevated SLC7A11 expression

**Figure 1:**
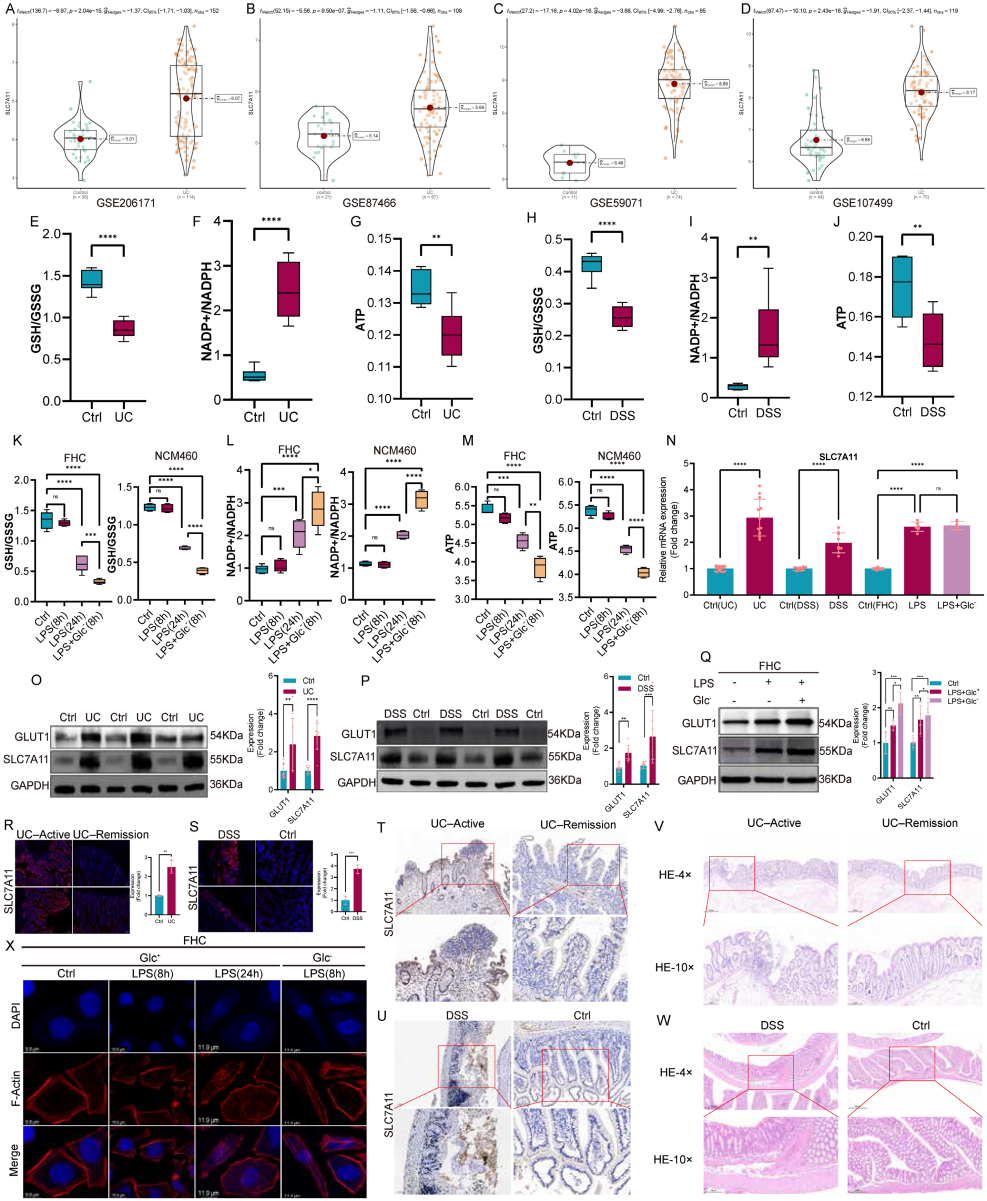
** Metabolic dysregulation and glucose deprivation characterize the UC intestine and are associated with elevated SLC7A11 expression. (A-D)** Violin plots showing SLC7A11 expression in colonic tissues from healthy controls and patients with ulcerative colitis across four independent transcriptomic datasets (GSE206171, GSE87466, GSE59071, and GSE107499). **(E-G)** Biochemical analyses of redox and energy metabolism in colonic tissues from control subjects and UC patients, including the GSH/GSSG ratio (E), NADP⁺/NADPH ratio (F), and ATP levels (G). **(H-J)** Corresponding metabolic parameters measured in colonic tissues from control mice and DSS-induced colitis mice, including the GSH/GSSG ratio (H), NADP⁺/NADPH ratio (I), and ATP levels (J). **(K-M)** Metabolic alterations in FHC and NCM460 intestinal epithelial cells treated with lipopolysaccharide (LPS, 8 h or 24 h) under glucose-replete or glucose-deprived conditions, as assessed by the GSH/GSSG ratio (K), NADP⁺/NADPH ratio (L), and ATP levels (M).** (N)** Relative mRNA expression of SLC7A11 in colonic tissues from UC patients and DSS-treated mice, as well as in FHC cells following LPS stimulation with or without glucose deprivation. **(O-Q)** Representative immunoblotting images and quantification of GLUT1 and SLC7A11 protein expression in colonic tissues from UC patients (O), DSS-induced colitis mice (P), and LPS-treated FHC cells under glucose-replete or glucose-deprived conditions (Q). **(R-S)** Immunofluorescence staining of SLC7A11 in colonic tissues from UC patients in active disease or remission (R), and from control or DSS-treated mice (S). **(T-U)** Representative immunohistochemical staining of SLC7A11 in colonic tissues from UC patients (T) and DSS-induced colitis mice (U). **(V-W)** Hematoxylin and eosin (H&E) staining of colonic tissues from UC patients (V) and DSS-induced colitis mice (W), illustrating histopathological alterations associated with intestinal inflammation. (X) Phalloidin staining of filamentous actin (F-actin) in FHC cells under control conditions, LPS stimulation, or glucose-deprived conditions, showing minimal changes in cell morphology following LPS treatment alone but pronounced cytoskeletal alterations under glucose deprivation. Data are presented as mean ± SEM. Statistical significance was determined using unpaired two-tailed Student's t-test or one-way ANOVA with appropriate post hoc tests. P values are indicated in the figures.

Analysis of multiple independent transcriptomic datasets revealed a consistent upregulation of SLC7A11 in intestinal tissues from patients with ulcerative colitis compared with healthy controls (Fig. 1A-D). To assess the metabolic landscape of the UC intestine, we examined redox and energy-related parameters. UC tissues exhibited a marked reduction in the GSH/GSSG ratio, accompanied by an increased NADP⁺/NADPH ratio and decreased ATP levels, indicative of impaired redox buffering capacity and energetic stress (Fig. 1E-G). Similar metabolic alterations were observed in DSS-induced colitis models (Fig. 1H-J). *In vitro*, inflammatory stimulation with LPS induced SLC7A11 expression in intestinal epithelial cells (FHC and NCM460), an effect that was further amplified under glucose-deprived conditions and associated with exacerbated redox imbalance (Fig. 1K-Q). Immunofluorescence and immunohistochemistry analyses confirmed elevated SLC7A11 expression in inflamed epithelial regions in both human UC samples and DSS-treated mice (Fig. 1R-U), coinciding with severe histopathological damage (Fig. 1V-W). Together, these data indicate that UC is characterized by profound metabolic stress and glucose deprivation, within which SLC7A11 is robustly induced. To mimic the glucose-deprived microenvironment observed in the UC intestine, intestinal epithelial cells were cultured under glucose-free conditions.

Phalloidin staining revealed that LPS stimulation alone caused minimal alterations in F-actin organization, whereas glucose deprivation rapidly induced pronounced cytoskeletal remodeling (Fig. 1X). Consistent with these findings, additional metabolic profiling further confirmed that glucose deprivation induces significant redox imbalance and energetic stress, as reflected by decreased GSH/GSSG ratio, increased NADP⁺/NADPH ratio, reduced ATP levels, and elevated ROS accumulation. These effects were reproducible in both FHC and NCM460, supporting the robustness of the observed metabolic phenotype (Supplementary Fig. S1). Given the established link between disulfide stress and actin cytoskeleton collapse, these findings suggest that glucose deprivation renders intestinal epithelial cells structurally vulnerable to disulfide stress-associated cell death programs.

### SLC7A11 exerts glucose-dependent dual effects under inflammatory stress

**Figure 2:**
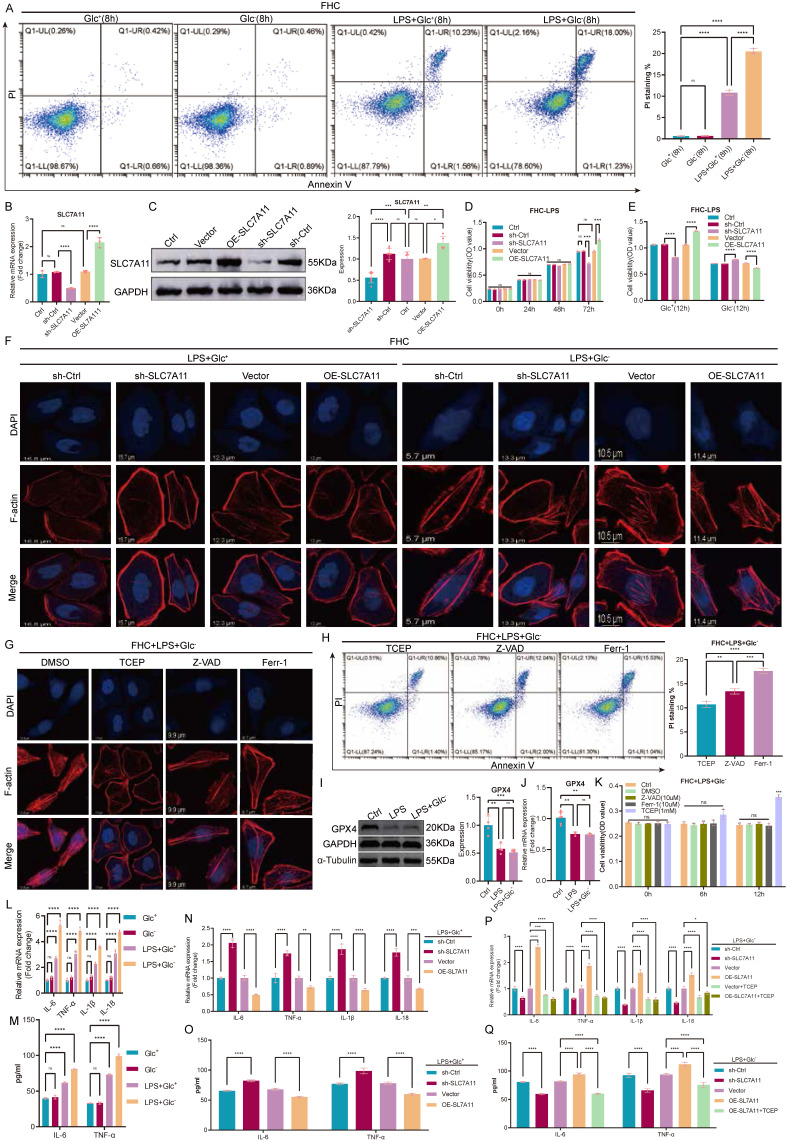
** SLC7A11 exhibits glucose-dependent dual effects on epithelial survival and inflammatory responses under inflammatory stress. (A)** Flow cytometric analysis of cell death in FHC cells treated with LPS under glucose-replete (Glc⁺) or glucose-deprived (Glc⁻) conditions using Annexin V and propidium iodide (PI) staining, with quantification of PI-positive cells shown on the right. **(B)** Relative mRNA expression of SLC7A11 in FHC cells following lentiviral-mediated knockdown or overexpression. **(C)** Representative immunoblotting images and quantification of SLC7A11 protein expression in control, knockdown, and overexpression FHC cells. **(D-E)** Cell viability assays showing the effects of SLC7A11 knockdown or overexpression on FHC cell survival following LPS stimulation under glucose-replete (D) or glucose-deprived (E) conditions. **(F)** Representative phalloidin staining of filamentous actin (F-actin) in FHC cells with SLC7A11 knockdown or overexpression following LPS stimulation under glucose-replete or glucose-deprived conditions. **(G)** Representative immunofluorescence images of F-actin organization in glucose-deprived, LPS-treated FHC cells following treatment with DMSO, the disulfide stress-reducing agent TCEP, the apoptosis inhibitor Z-VAD-FMK, or the ferroptosis inhibitor Ferrostatin-1. **(H)** Flow cytometric analysis and quantification of PI-positive cells in glucose-deprived, LPS-treated FHC cells following pharmacological inhibition of distinct cell death pathways. **(I)** Representative immunoblotting images of GPX4 expression in FHC cells treated with LPS under glucose-replete or glucose-deprived conditions. **(J)** Relative mRNA expression of GPX4 under the indicated conditions. **(K)** Cell viability analysis of glucose-deprived, LPS-treated FHC cells following treatment with DMSO, Z-VAD-FMK, Ferrostatin-1, or TCEP. **(L-M)** Relative mRNA expression (L) and ELISA quantification (M) of pro-inflammatory cytokines in FHC cells treated with LPS under glucose-replete or glucose-deprived conditions without genetic manipulation of SLC7A11. **(N-O)** Relative mRNA expression (N) and ELISA quantification (O) of pro-inflammatory cytokines in FHC cells treated with LPS under glucose-replete conditions following SLC7A11 knockdown or overexpression. **(P-Q)** Effects of SLC7A11 knockdown or overexpression, with or without TCEP treatment, on inflammatory cytokine expression at the mRNA (P) and protein (Q) levels in glucose-deprived, LPS-treated FHC cells. Data are presented as mean ± SEM. Statistical significance was determined using unpaired two-tailed Student's t-test or one-way ANOVA with appropriate post hoc tests. P values are indicated in the figures.

Inflammatory stimulation robustly increased epithelial cell death under glucose-deprived conditions. Flow cytometric analysis using Annexin V/propidium iodide staining showed that LPS treatment induced only limited cell death in glucose-replete medium, whereas glucose deprivation markedly sensitized FHC cells to LPS-induced death, leading to a pronounced increase in PI-positive cells (Fig. 2A). To causally define the role of SLC7A11 in this context, we generated stable FHC cell lines with lentiviral SLC7A11 knockdown or overexpression, and validated modulation efficiency at both the mRNA and protein levels (Fig. 2B-C). Under glucose-replete conditions, SLC7A11 overexpression improved cell viability following LPS stimulation, while SLC7A11 knockdown reduced viability, consistent with a cytoprotective role when glucose supply is sufficient (Fig. 2D-E). In contrast, under glucose-deprived conditions, SLC7A11 overexpression significantly aggravated cell death and loss of viability after LPS challenge, whereas SLC7A11 knockdown partially mitigated these effects (Fig. 2A, E). We next examined whether the divergent survival outcomes were associated with cytoskeletal integrity. Phalloidin staining revealed that LPS stimulation alone caused minimal changes in F-actin organization in glucose-replete medium. However, glucose deprivation led to rapid and striking F-actin remodeling and cytoskeletal disruption, which was exacerbated by SLC7A11 overexpression and alleviated by SLC7A11 knockdown (Fig. 2F). To delineate the dominant cell death program involved, LPS-treated, glucose-deprived FHC cells were treated with inhibitors targeting apoptosis (Z-VAD-FMK), ferroptosis (Ferrostatin-1), or disulfide stress (TCEP). Among these interventions, TCEP effectively restored F-actin organization and reduced cell death, whereas Z-VAD-FMK and Ferrostatin-1 provided minimal protection (Fig. 2G-H). Consistently, GPX4 expression remained largely unchanged at both the protein and transcript levels under glucose-deprived conditions compared with glucose-replete controls (Fig. 2I-J), supporting that the glucose deprivation-associated death phenotype was largely independent of GPX4-mediated ferroptosis.

Finally, we assessed inflammatory outputs under these conditions. Glucose deprivation markedly enhanced LPS-induced expression of pro-inflammatory cytokines (IL-6, TNF-α, IL-1β, and IL-18) at the mRNA level (Fig. 2L-N), and increased secretion of IL-6 and TNF-α at the protein level (Fig. 2M, O). Notably, SLC7A11 overexpression further amplified cytokine production under glucose deprivation, whereas SLC7A11 knockdown or TCEP treatment attenuated these inflammatory responses (Fig. 2P-Q). Together, these findings indicate that inflammatory stimulation induces SLC7A11 expression across metabolic contexts, but glucose availability dictates its functional outcome: SLC7A11 is cytoprotective and limits inflammatory damage when glucose is sufficient, yet under glucose deprivation it promotes cytoskeleton-associated, disulfide stress-linked epithelial cell death and amplifies inflammatory responses.

### METTL1 mediates m7G modification of SLC7A11 mRNA and enhances its transcript stability

**Figure 3:**
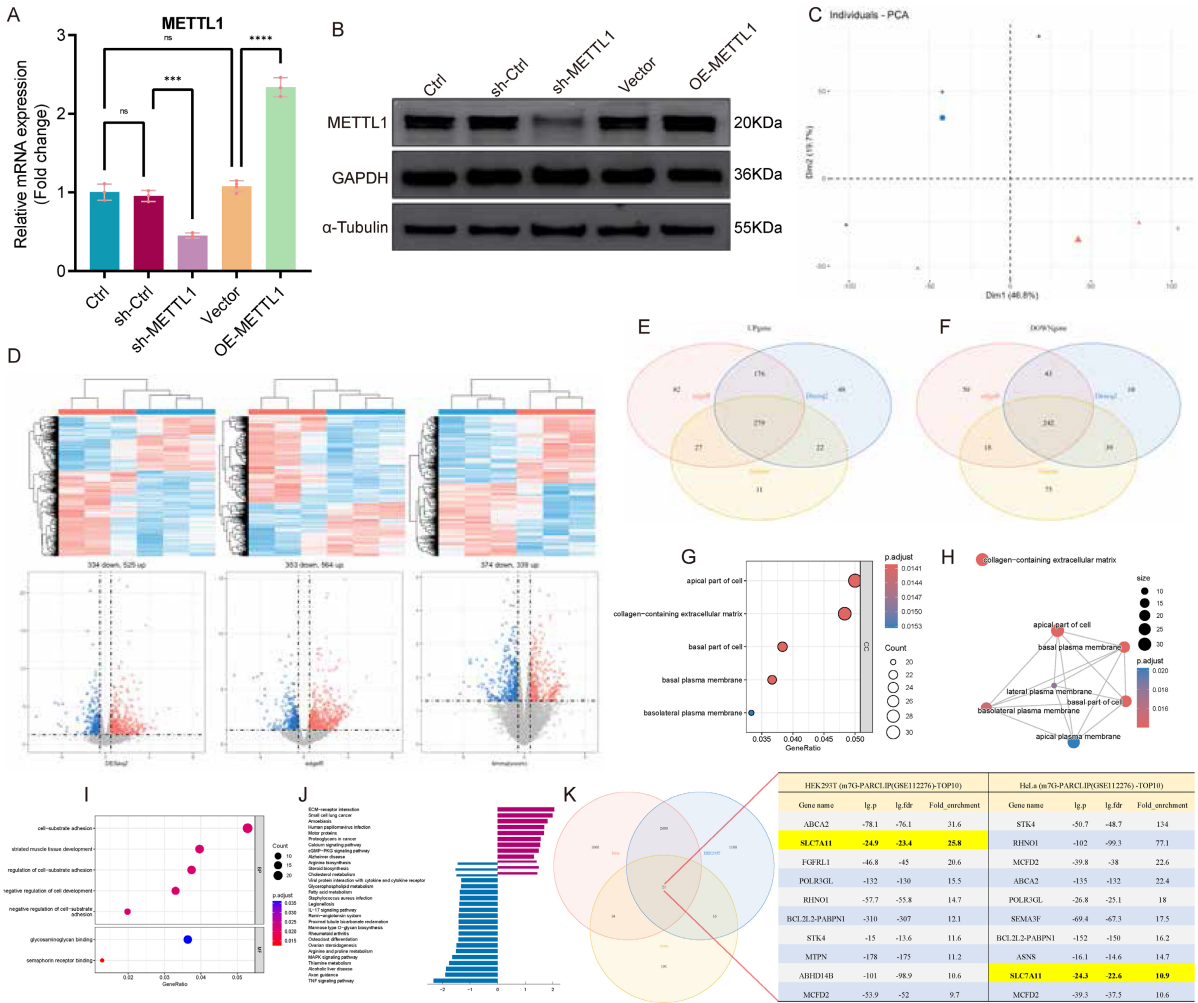
** Transcriptome-wide identification of SLC7A11 as a METTL1-associated m7G-modified target. (A)** Relative mRNA expression of METTL1 in FHC cells following lentiviral-mediated knockdown or overexpression. **(B)** Representative immunoblotting images and quantification of METTL1 protein expression under METTL1 knockdown or overexpression conditions. **(C)** Principal component analysis (PCA) of RNA-seq data showing clear separation between METTL1 knockdown and control FHC cells (n = 3 per group). **(D)** Heatmap and volcano plot illustrating differentially expressed genes identified from RNA-seq analysis following METTL1 knockdown using three independent methods (DESeq2, edgeR, and limma) under the same cutoff criteria (|log₂FC| > 1, P < 0.05). **(E)** Venn diagram showing the overlap of differentially expressed genes identified by DESeq2, edgeR, and limma. **(F)** Numbers of commonly upregulated and downregulated genes identified by the intersection of the three analytical approaches. **(G)** GO enrichment (Cellular Component) bubble plot showing significant enrichment in apical, basal, lateral, and basolateral plasma membrane components and collagen-containing extracellular matrix. Bubble size = gene count; color = adjusted p-value; x-axis = GeneRatio. **(H)** Network of enriched GO Cellular Component terms. Node size = gene count; node color = adjusted p-value; edges = shared genes. Plasma-membrane-related terms form a tightly connected cluster. **(I)** GO enrichment (Biological Process / Molecular Function) bubble plots highlighting cell-substrate adhesion, regulation of adhesion, negative regulation of cell development, and extracellular-matrix binding activities. **(J)** KEGG pathway enrichment bar plot showing downregulation of lipid-metabolism pathways (glycerophospholipid, fatty-acid, cholesterol metabolism) and perturbations in MAPK, TNF, and IL-17 signaling. **(K)** Intersection analysis between the METTL1-regulated differentially expressed genes and an m7G-enriched gene set derived from a public m7G-seq dataset (GSE112276), identifying SLC7A11 as a candidate METTL1-associated m7G-modified target. Data are presented as mean ± SEM unless otherwise indicated. Statistical significance thresholds for RNA-seq analyses are described in the Methods.

Based on our previous findings that m7G methylation is closely associated with intestinal inflammation in UC 16, we focused on METTL1, a core m7G methyltransferase, to systematically identify downstream targets. We first established stable METTL1 knockdown and overexpression cell lines via lentiviral transduction and verified manipulation efficiency at both the mRNA (Fig. 3A) and protein levels (Fig. 3B).

RNA-seq was then performed comparing METTL1 knockdown cells with controls (n = 3 per group), and principal component analysis demonstrated clear separation between groups (Fig. 3C). For differential expression, DESeq2, edgeR, and limma were applied in parallel under the same criteria (|log₂FC| > 1, P < 0.05), and global expression changes were visualized using heatmaps and volcano plots (Fig. 3D). Intersection of differentially expressed genes identified by the three methods yielded 279 upregulated and 242 downregulated genes, supporting the robustness of the METTL1-dependent transcriptional signature (Fig. 3E-F). GO and KEGG enrichment analyses indicated that these genes were predominantly involved in redox homeostasis, amino acid transport, and cellular stress responses, consistent with the known biological functions of SLC7A11 (Fig. 3G-J). To further prioritize potential m7G-modified targets among METTL1-regulated genes, we retrieved a public m7G-seq dataset (GSE112276) and intersected the m7G-enriched gene set with our differentially expressed genes. Notably, the overlap analysis and candidate list highlighted SLC7A11 as a prominent candidate, suggesting a close association with METTL1-mediated m7G-dependent regulation (Fig. 3K).

**Figure 4:**
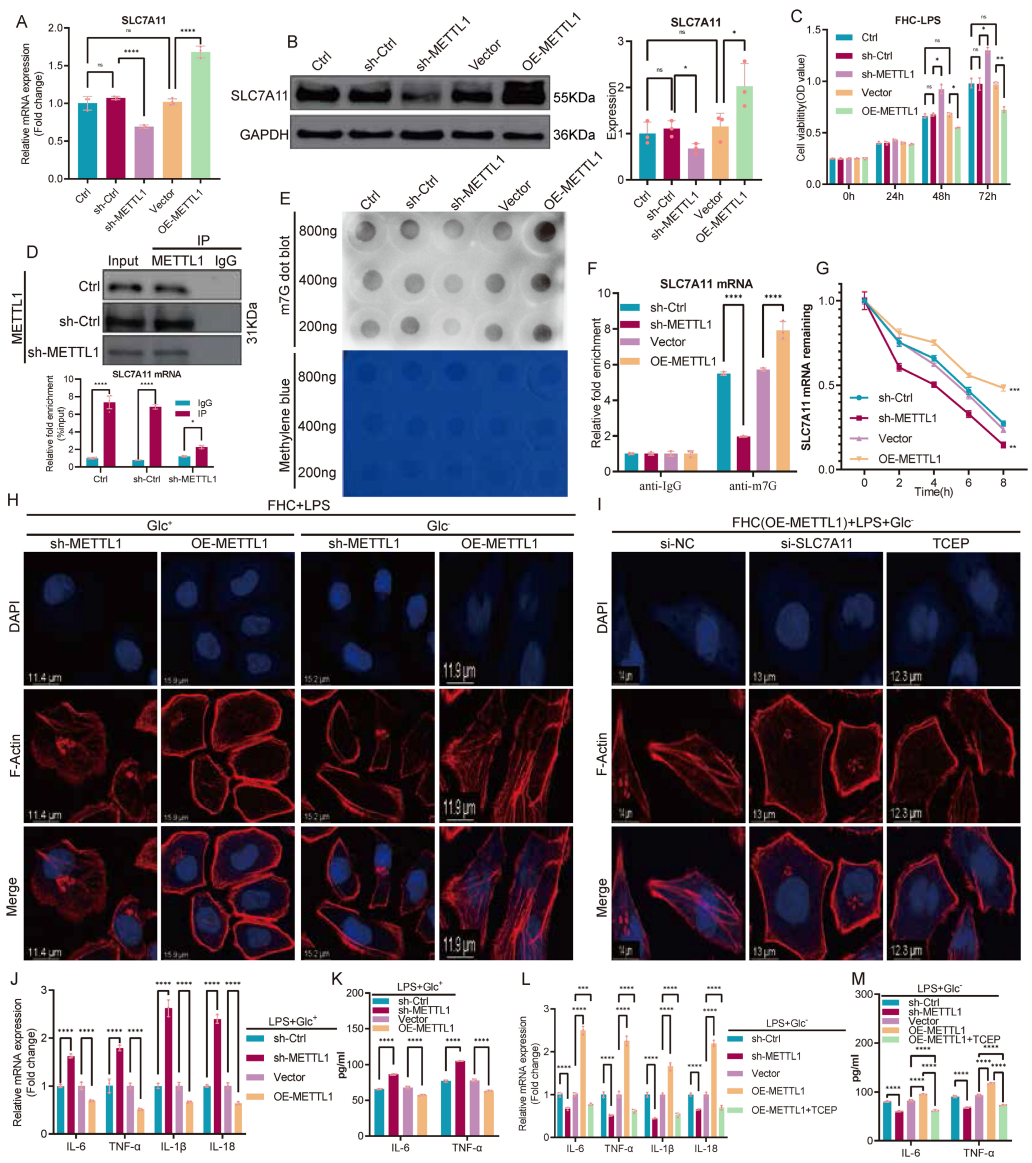
** METTL1 enhances m7G modification and stabilizes SLC7A11 mRNA, thereby regulating epithelial fate under metabolic stress. (A)** Relative mRNA expression of SLC7A11 in FHC cells following METTL1 knockdown or overexpression. **(B)** Representative immunoblotting images and quantification of SLC7A11 protein expression under METTL1 knockdown or overexpression conditions. **(C)** Cell viability analysis of FHC cells following LPS stimulation under METTL1 knockdown or overexpression, assessed at the indicated time points. **(D)** RNA immunoprecipitation (RIP) assays showing enrichment of SLC7A11 mRNA in METTL1 immunoprecipitates compared with IgG controls. **(E)** Dot blot analysis of global m7G modification levels in FHC cells following METTL1 knockdown or overexpression, with methylene blue staining shown as a loading control. **(F)** m7G RNA immunoprecipitation (MeRIP)-qPCR analysis showing enrichment of SLC7A11 mRNA in anti-m7G immunoprecipitates under METTL1 knockdown or overexpression conditions. **(G)** Actinomycin D chase assays showing the decay kinetics of SLC7A11 mRNA in FHC cells with METTL1 knockdown or overexpression. **(H)** Phalloidin staining of filamentous actin (F-actin) in FHC cells with METTL1 knockdown or overexpression following LPS stimulation under glucose-replete (Glc⁺) or glucose-deprived (Glc⁻) conditions. **(I)** Representative F-actin staining in METTL1-overexpressing FHC cells following SLC7A11 knockdown or treatment with the disulfide stress-reducing agent TCEP under glucose-deprived and LPS-stimulated conditions. **(J)** Relative mRNA expression of inflammatory cytokines in FHC cells with METTL1 knockdown or overexpression following LPS stimulation under glucose-deprived conditions. **(K)** ELISA quantification of inflammatory cytokine secretion in culture supernatants under the indicated conditions. **(L)** Relative mRNA expression of inflammatory cytokines in METTL1-overexpressing FHC cells treated with or without TCEP under glucose-deprived and LPS-stimulated conditions. **(M)** ELISA quantification of inflammatory cytokine secretion in METTL1-overexpressing FHC cells with or without TCEP treatment under glucose-deprived and LPS-stimulated conditions. Data are presented as mean ± SEM. Statistical significance was determined using unpaired two-tailed Student's t-test or one-way ANOVA with appropriate post hoc tests. P values are indicated in the figures.

Following the identification of SLC7A11 as a potential downstream target of METTL1, we next performed a systematic validation of the METTL1-SLC7A11 regulatory mechanism. Manipulation of METTL1 expression in FHC cells revealed that SLC7A11 mRNA levels changed concordantly with METTL1 expression, which was further confirmed at the protein level (Fig. 4A-B). Functionally, either knockdown or overexpression of METTL1 significantly altered cell viability following LPS stimulation (Fig. 4C). To determine whether METTL1 directly regulates SLC7A11 transcripts, we performed RNA immunoprecipitation (RIP) assays. SLC7A11 mRNA was markedly enriched in METTL1 immunoprecipitates compared with IgG controls, whereas this enrichment was substantially reduced upon METTL1 knockdown (Fig. 4D), indicating a specific association between METTL1 and SLC7A11 mRNA. Consistently, dot blot analysis showed that global m7G modification levels were significantly decreased by METTL1 knockdown and increased by METTL1 overexpression (Fig. 4E). Further m7G RNA immunoprecipitation (MeRIP) followed by qPCR demonstrated robust enrichment of SLC7A11 mRNA in anti-m7G immunoprecipitates, which was attenuated upon METTL1 depletion and enhanced upon METTL1 overexpression (Fig. 4F). To assess whether m7G modification affects transcript stability, transcription was blocked using actinomycin D, and mRNA decay kinetics were analyzed. METTL1 knockdown significantly accelerated SLC7A11 mRNA degradation, whereas METTL1 overexpression markedly delayed transcript decay (Fig. 4G), indicating that METTL1 enhances SLC7A11 mRNA stability in an m7G-dependent manner. At the phenotypic level, phalloidin staining revealed that, under LPS stimulation, METTL1 overexpression markedly exacerbated F-actin disruption under glucose-deprived conditions, whereas METTL1 knockdown partially preserved cytoskeletal integrity (Fig. 4H). Notably, in the METTL1-overexpressing background, either SLC7A11 silencing or treatment with the disulfide bond-reducing agent TCEP substantially alleviated F-actin disorganization (Fig. 4I). In addition, METTL1 knockdown or overexpression significantly modulated inflammatory cytokine expression at the transcript level (Fig. 4J) and resulted in corresponding changes in cytokine secretion (Fig. 4K). Under combined glucose deprivation and LPS stimulation, TCEP treatment effectively suppressed the inflammatory amplification induced by METTL1 overexpression (Fig. 4L-M). Collectively, these results indicate that METTL1 enhances m7G modification and stabilizes SLC7A11 mRNA, thereby promoting SLC7A11 expression and regulating epithelial structural integrity, cell fate, and inflammatory responses under metabolic stress.

### METTL1-mediated m7G modification is upregulated in ulcerative colitis

**Figure 5:**
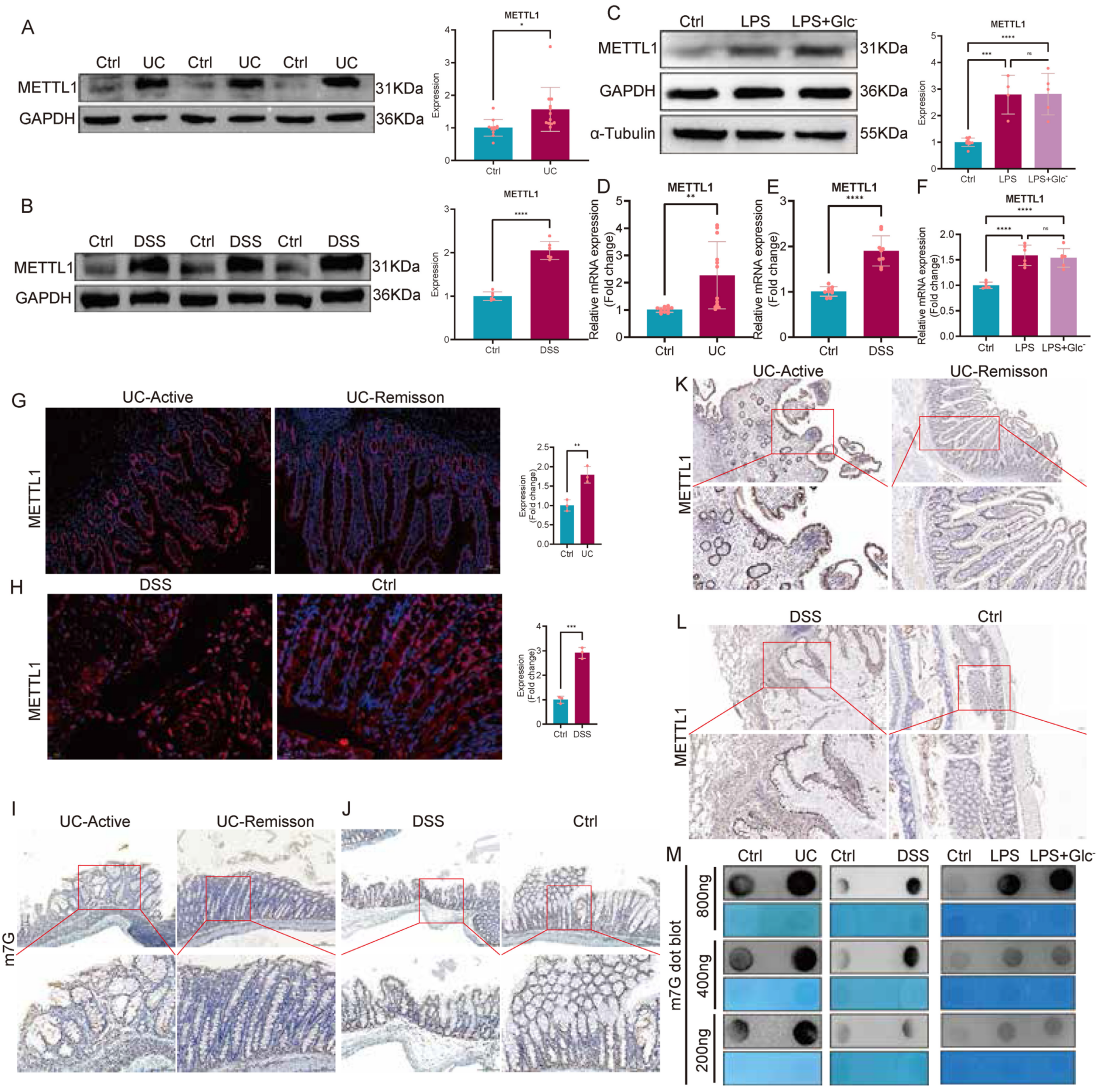
** METTL1 expression and METTL1-mediated m7G modification are upregulated in ulcerative colitis. (A)** Representative immunoblotting images and quantification of METTL1 protein expression in intestinal tissues from patients with ulcerative colitis (UC) and healthy controls. **(B)** Immunoblotting analysis and quantification of METTL1 protein expression in colonic tissues from DSS-induced colitis mice and control mice. **(C)** Representative immunoblotting images and quantification of METTL1 protein expression in FHC cells treated with LPS or LPS under glucose-deprived conditions. **(D)** Relative mRNA expression of METTL1 in intestinal tissues from UC patients and healthy controls. **(E)** Relative mRNA expression of METTL1 in colonic tissues from DSS-treated mice and control mice. **(F)** Relative mRNA expression of METTL1 in FHC cells following LPS stimulation with or without glucose deprivation. **(G)** Representative immunofluorescence staining of METTL1 in intestinal tissues from patients with active UC and UC in remission, with quantification shown on the right. **(H)** Representative immunofluorescence staining of METTL1 in colonic tissues from DSS-treated mice and control mice, with quantification shown on the right. **(I,J)** Immunohistochemical staining of m7G modification in intestinal tissues from patients with active UC and UC in remission (I), as well as in DSS-treated mice and controls (J). **(K)** Immunohistochemical staining of METTL1 in intestinal tissues from patients with active UC and UC in remission. **(L)** Immunohistochemical staining of METTL1 in colonic tissues from DSS-treated mice and control mice. **(M)** Dot blot analysis of global m7G modification levels in intestinal tissues from UC patients, DSS-induced colitis mice, and FHC cells treated with LPS or LPS under glucose-deprived conditions, with methylene blue staining shown as a loading control. Data are presented as mean ± SEM. Statistical significance was determined using unpaired two-tailed Student's t-test or one-way ANOVA with appropriate post hoc tests. P values are indicated in the figures.

To further characterize the expression pattern of METTL1 and METTL1-mediated m7G modification in UC, we first examined METTL1 expression in intestinal tissues from patients with ulcerative colitis. Western blot analysis revealed a significant increase in METTL1 protein levels in UC tissues compared with controls (Fig. 5A), which was similarly observed in DSS-induced colitis mice (Fig. 5B). Consistently, qPCR analysis confirmed that METTL1 mRNA expression was markedly elevated in both UC patients and DSS-treated mice (Fig. 5D-E). *In vitro*, inflammatory stimulation with LPS significantly induced METTL1 expression in FHC cells, and this effect was further enhanced under glucose-deprived conditions (Fig. 5C, F), suggesting a synergistic effect of inflammatory and metabolic stress on METTL1 upregulation. To define the spatial distribution of METTL1 in intestinal tissues, immunofluorescence and immunohistochemical analyses were performed. METTL1 expression was markedly increased in the epithelial compartment of active UC tissues, whereas reduced expression was observed in remission samples (Fig. 5G, K). Similarly, in DSS-induced colitis mice, METTL1 was prominently enriched in inflamed colonic regions (Fig. 5H, L). Given that METTL1 is a core m7G methyltransferase, we further assessed global m7G modification levels in intestinal tissues. Immunohistochemical staining demonstrated enhanced m7G signals in active UC tissues and DSS-treated mice compared with controls (Fig. 5I, J). Moreover, dot blot analysis confirmed a robust increase in global m7G modification levels in UC tissues, DSS mouse colons, and LPS-stimulated FHC cells (Fig. 5M). Collectively, these findings indicate that METTL1 expression and METTL1-mediated m7G modification are consistently upregulated in UC-associated inflammatory settings, spanning human samples, animal models, and *in vitro* inflammatory conditions.

### The METTL1/m7G/SLC7A11 axis attenuates chronic DSS-induced colitis

**Figure 6:**
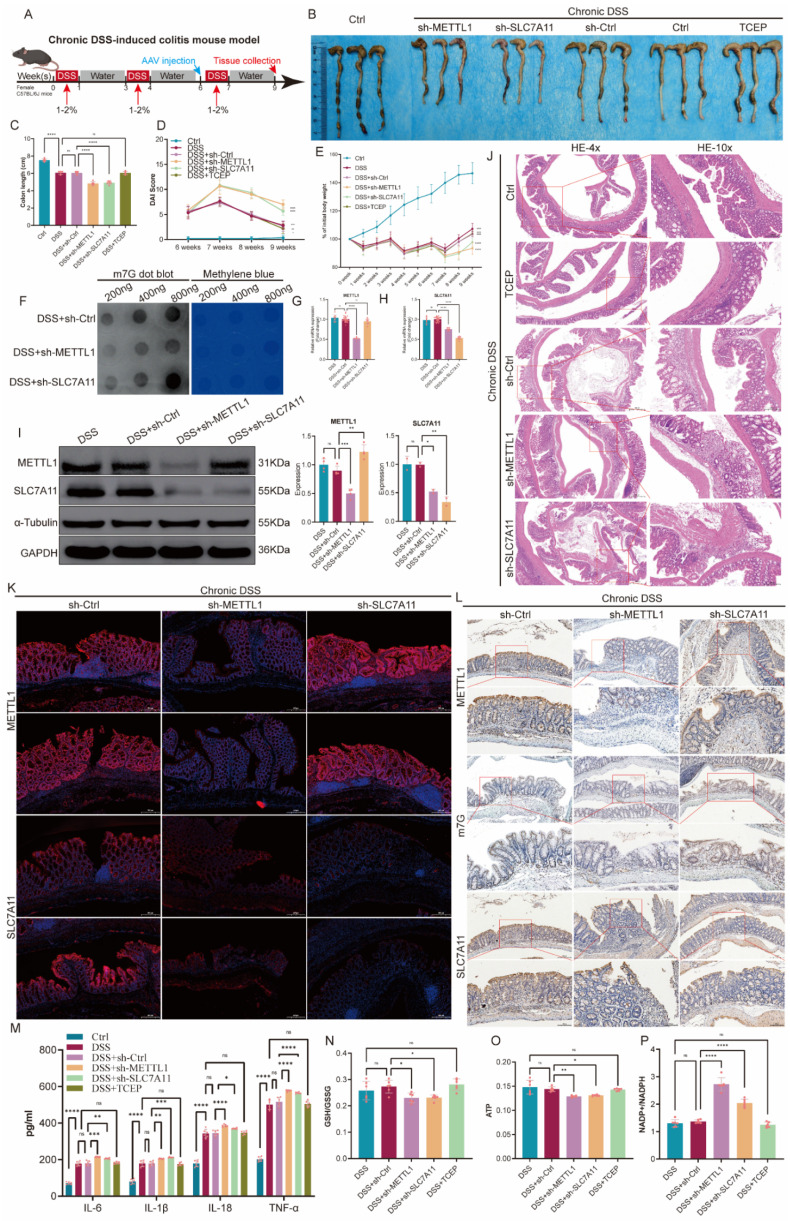
** Inhibition of the METTL1/m7G/SLC7A11 axis aggravates chronic DSS-induced colitis. (A)** Schematic illustration of the experimental design for the chronic DSS-induced colitis mouse model, including DSS administration schedule, adeno-associated virus (AAV), and tissue collection. **(B)** Representative images of colons isolated from control mice and mice subjected to chronic DSS treatment with sh-Ctrl, sh-METTL1, sh-SLC7A11, or TCEP treatment. **(C)** Quantification of colon length in the indicated groups. **(D)** Longitudinal assessment of disease activity index (DAI) during chronic DSS exposure. **(E)** Body weight changes of mice throughout the chronic DSS treatment period. **(F)** Dot blot analysis of global m7G modification levels in colonic tissues from chronic DSS-treated mice with METTL1 or SLC7A11 knockdown, with methylene blue staining shown as a loading control. **(G-H)** Relative mRNA expression levels of METTL1 (G) and SLC7A11 (H) in colonic tissues from the indicated groups. **(I)** Representative immunoblotting images and quantification of METTL1 and SLC7A11 protein expression in colonic tissues. **(J)** Representative hematoxylin and eosin (HE) staining of colonic sections from the indicated groups (low and high magnification). **(K)** Representative immunofluorescence staining of METTL1 and SLC7A11 in colonic tissues from mice subjected to chronic DSS treatment with the indicated interventions. **(L)** Representative immunohistochemical staining of METTL1, m7G modification, and SLC7A11 in colonic tissues from the indicated groups. **(M)** Levels of inflammatory cytokines in colonic tissues from the indicated groups. **(N-P)** Assessment of redox balance and energy metabolism, including GSH/GSSG ratio (N), ATP levels (O), and NADP⁺/NADPH ratio (P), in colonic tissues from the indicated groups. Data are presented as mean ± SEM. Statistical significance was determined using one-way ANOVA with appropriate post hoc tests. P values are indicated in the figures.

To interrogate the function of the METTL1/m7G/SLC7A11 axis under a mild and sustained inflammatory environment, rather than severe inflammation-associated metabolic collapse and glucose deprivation, we established a chronic DSS-induced colitis mouse model. During disease induction, AAV-mediated knockdown of METTL1 or SLC7A11 was performed, and TCEP treatment was included as a pharmacological perturbation (Fig. 6A). At the macroscopic level, chronic DSS administration caused significant body weight loss, colon shortening, and increased disease activity index (DAI). Notably, knockdown of METTL1 or SLC7A11 further aggravated DSS-induced disease manifestations, as evidenced by more pronounced weight loss, more severe colon shortening, and persistently higher DAI scores compared with DSS controls (Fig. 6B-E). Consistently, TCEP treatment also exacerbated clinical parameters during chronic DSS exposure (Fig. 6B-E). At the molecular level, chronic DSS colons exhibited increased METTL1 and SLC7A11 expression together with elevated global m7G modification levels. As expected, METTL1 knockdown reduced METTL1 expression and lowered global m7G signals, accompanied by decreased SLC7A11 expression, whereas SLC7A11 knockdown selectively reduced SLC7A11 levels (Fig. 6F-I). Despite these molecular changes, suppression of this axis was associated with worsened disease phenotypes in the chronic setting. Histological examination further supported the protective role of this axis. Hematoxylin and eosin (HE) staining showed epithelial disruption, crypt damage, and inflammatory infiltration in DSS-treated mice, all of which were more severe upon METTL1 or SLC7A11 knockdown (Fig. 6J). Immunofluorescence and immunohistochemical analyses confirmed epithelial enrichment of METTL1 and SLC7A11 and elevated m7G signals in chronic DSS colitis, whereas the corresponding signals were reduced in the knockdown groups (Fig. 6K-L). At the inflammatory and metabolic levels, chronic DSS exposure induced cytokine production and metabolic imbalance, including reduced GSH/GSSG ratios, decreased ATP levels, and increased NADP⁺/NADPH ratios. Importantly, METTL1 or SLC7A11 knockdown further intensified these inflammatory and metabolic disturbances during chronic DSS treatment, consistent with aggravated mucosal injury. Collectively, these findings indicate that, under chronic DSS-induced colitis, the METTL1/m7G/SLC7A11 axis exerts a protective, inflammation-limiting effect, and genetic or pharmacological suppression of this axis exacerbates disease severity.

### Inhibition of the METTL1/m7G/SLC7A11 axis alleviates acute DSS-induced colitis

**Figure 7:**
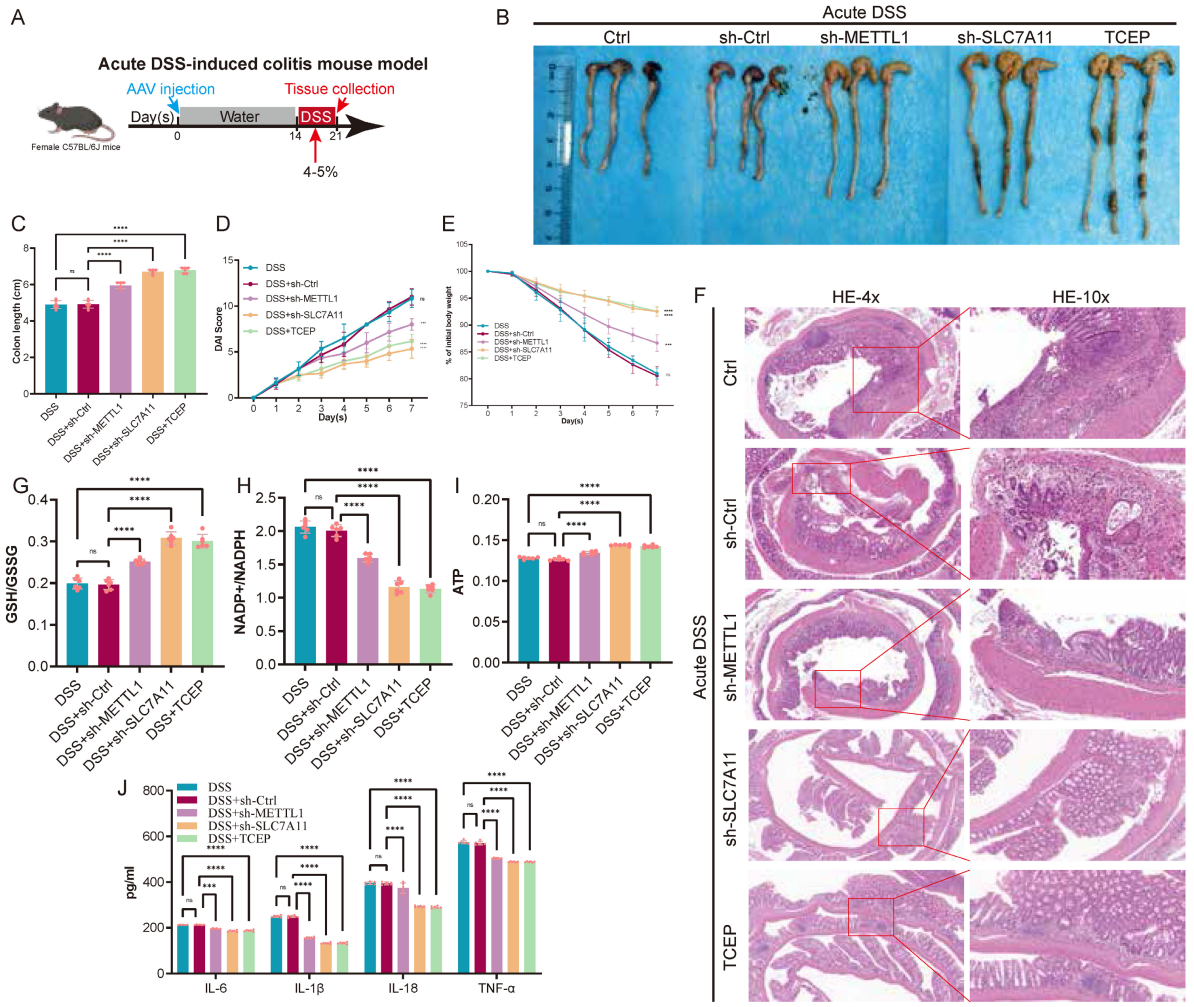
** Inhibition of the METTL1/m7G/SLC7A11 axis alleviates acute DSS-induced colitis. (A)** Schematic illustration of the experimental design for the acute DSS-induced colitis mouse model, including DSS administration, AAV injection, and tissue collection. **(B)** Representative images of colons isolated from control mice and mice subjected to acute DSS treatment with sh-Ctrl, sh-METTL1, sh-SLC7A11, or TCEP treatment. **(C)** Quantification of colon length in the indicated groups. **(D)** Disease activity index (DAI) scores monitored during acute DSS exposure. **(E)** Body weight changes of mice throughout the acute DSS treatment period. **(F)** Representative hematoxylin and eosin (HE) staining of colonic sections from the indicated groups (low and high magnification). **(G-I)** Assessment of redox balance and energy metabolism in colonic tissues, including GSH/GSSG ratio (G), NADP⁺/NADPH ratio (H), and ATP levels (I). **(J)** Levels of inflammatory cytokines in colonic tissues from the indicated groups. Data are presented as mean ± SEM. Statistical significance was determined using one-way ANOVA with appropriate post hoc tests. P values are indicated in the figures.

To determine the role of the METTL1/m7G/SLC7A11 axis during acute intestinal inflammation, we established an acute DSS-induced colitis mouse model, which is characterized by rapid disease progression accompanied by severe metabolic stress. During DSS exposure, AAV-mediated knockdown of METTL1 or SLC7A11 was performed, and TCEP treatment was included as a pharmacological intervention (Fig. 7A). At the macroscopic level, acute DSS administration resulted in pronounced colon shortening, body weight loss, and elevated DAI. Notably, knockdown of METTL1 or SLC7A11 significantly alleviated acute disease severity, as reflected by increased colon length, attenuated weight loss, and reduced DAI scores compared with DSS control mice (Fig. 7B-E). Consistently, TCEP treatment also markedly improved these clinical parameters. Histological examination revealed extensive epithelial damage, crypt loss, and inflammatory infiltration in DSS-treated colons. In contrast, METTL1 or SLC7A11 knockdown substantially preserved mucosal architecture, with reduced epithelial disruption and inflammatory cell infiltration. A similar protective effect was observed in TCEP-treated mice (Fig. 7F). At the metabolic level, acute DSS challenge induced profound redox imbalance and energy depletion, as evidenced by decreased GSH/GSSG ratios, increased NADP⁺/NADPH ratios, and reduced ATP levels. Importantly, suppression of METTL1 or SLC7A11 significantly restored redox homeostasis and ATP content, whereas DSS control mice exhibited persistent metabolic dysfunction (Fig. 7G-I). In parallel, acute DSS exposure robustly induced inflammatory cytokine production. In line with the improved disease phenotype, METTL1 or SLC7A11 knockdown markedly reduced cytokine levels, and TCEP treatment exerted a comparable anti-inflammatory effect (Fig. 7J). Collectively, these results demonstrate that, under acute DSS-induced colitis associated with severe metabolic stress, inhibition of the METTL1/m7G/SLC7A11 axis alleviates intestinal inflammation and tissue injury, highlighting a context-dependent, maladaptive role of this axis during acute disease.

## Discussion

**Figure 8:**
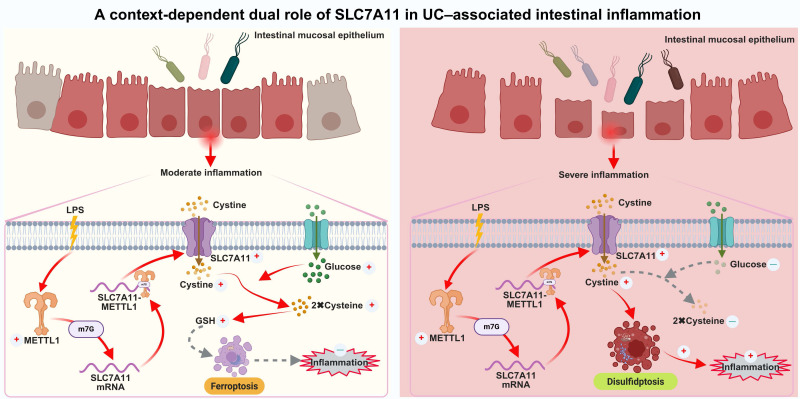
** Proposed model illustrating the context-dependent dual role of the METTL1-m7G-SLC7A11 axis in UC-associated intestinal inflammation.** Under moderate and sustained inflammatory conditions, intestinal epithelial cells retain relatively sufficient glucose availability. In this context, inflammatory stimulation induces METTL1 expression and enhances METTL1-mediated m7G modification of SLC7A11 mRNA, thereby stabilizing the transcript and promoting SLC7A11 expression. Elevated SLC7A11 facilitates cystine uptake and its reduction to cysteine, supporting glutathione (GSH) synthesis, maintaining redox homeostasis, and suppressing ferroptosis-associated epithelial injury, ultimately limiting inflammatory damage. In contrast, under severe inflammation accompanied by glucose deprivation and profound metabolic stress, persistent activation of the METTL1-m7G-SLC7A11 axis leads to excessive cystine uptake in a glucose-deficient environment. Impaired cystine reduction and redox imbalance result in disulfide stress, triggering cytoskeletal collapse and disulfidptosis in intestinal epithelial cells. This maladaptive response exacerbates epithelial damage and amplifies intestinal inflammation. Collectively, this model highlights the METTL1-m7G-SLC7A11 axis as a metabolic rheostat that integrates inflammatory signals and nutrient availability to determine epithelial cell fate in UC.

This study identifies a context-dependent METTL1-m7G-SLC7A11 regulatory axis that links metabolic stress to intestinal epithelial cell fate during the progression of UC. By integrating analyses of human UC tissues, DSS-induced colitis mouse models, and *in vitro* mechanistic experiments, we demonstrate that the functional effects of this axis are highly dependent on inflammatory intensity and the local metabolic environment (Fig. 8). Specifically, under mild and sustained inflammatory conditions, this axis exerts anti-inflammatory and protective effects, whereas during acute inflammation accompanied by severe metabolic dysregulation, it becomes maladaptive, promoting epithelial injury and amplifying inflammatory responses 24.

A central finding of this study is that SLC7A11 is a direct downstream target of METTL1-mediated m7G modification in intestinal epithelial cells. METTL1 binds specifically to SLC7A11 mRNA and enhances its m7G modification, thereby increasing transcript stability. This mechanism provides an epitranscriptomic basis for the persistent upregulation of SLC7A11 observed in UC tissues and experimental colitis models. Our findings extend the functional relevance of m7G modification to chronic inflammatory diseases and establish METTL1 as an important regulator of epithelial stress responses in the intestinal mucosa.

Previous studies have reported conflicting roles of SLC7A11 in inflammatory diseases. Several studies suggest that upregulation of SLC7A11 alleviates ulcerative colitis by suppressing ferroptosis in intestinal epithelial cells 25-29, whereas other work has shown that inhibition of SLC7A11 can enhance epithelial barrier function and ameliorate colitis 30. Our results further indicate that the functional divergence of SLC7A11 may be largely determined by inflammation-associated glucose availability. Mechanistically, the functional output of SLC7A11 is tightly constrained by cellular NADPH availability. SLC7A11 mediates cystine uptake, which must be reduced to cysteine for subsequent glutathione synthesis, a process that consumes reducing equivalents in the form of NADPH. Under glucose-replete conditions, sufficient NADPH is generated through the pentose phosphate pathway, enabling SLC7A11 to support antioxidant defense 14. However, under glucose deprivation, NADPH production is limited, and continued cystine influx leads to accumulation of disulfide stress. This metabolic imbalance shifts SLC7A11 function from maintaining redox homeostasis to promoting cytotoxic stress, thereby providing a mechanistic basis for the stage-dependent functional switch observed in our study. Under glucose-replete conditions, SLC7A11 supports cystine uptake and glutathione synthesis, thereby maintaining redox homeostasis and preserving epithelial structural integrity 31,32. In contrast, under glucose deprivation—a characteristic metabolic feature of inflamed UC mucosa—sustained SLC7A11 overexpression induces excessive disulfide stress, leading to F-actin collapse and epithelial injury, consistent with phenotypes associated with disulfidptosis. These findings provide a unifying framework to reconcile previous inconsistent observations regarding the role of SLC7A11 under different inflammatory and stress conditions. Studies have shown that acute and chronic DSS-induced mouse models can be used to mimic the acute severe phase and disease remission phase in the pathogenesis of UC 33. Our results indeed confirm the dual role of the METTL1/m7G/SLC7A11 axis in UC under varying degrees of disease severity.

Nevertheless, disulfidptosis is a recently described form of regulated cell death, and reliable, widely accepted molecular markers for its identification—particularly *in vivo*—remain limited. Although our study provides multiple lines of evidence, including NADPH depletion, redox imbalance, cytoskeletal collapse, lack of response to apoptosis and ferroptosis inhibitors, and sensitivity to disulfide-reducing agents, these findings should be interpreted as being consistent with, rather than definitively proving, disulfidptosis 7. Future studies incorporating more specific molecular markers, lineage tracing approaches, or high-resolution metabolic flux analyses will be required to more precisely delineate epithelial cell death programs in UC. In addition, while this work focuses on intestinal epithelial cells, interactions with immune and stromal compartments may further modulate the METTL1/m7G/SLC7A11 axis *in vivo* and warrant further investigation. Moreover, emerging artificial intelligence-based approaches may provide powerful tools for integrating multi-omics datasets and identifying complex cell death signatures 34, thereby facilitating more precise characterization of disulfidptosis and its role in UC pathogenesis.

In summary, this study supports a model in which the METTL1-m7G-SLC7A11 axis integrates nutrient availability and redox status to determine programmed epithelial cell death during UC progression. From a therapeutic perspective, these findings highlight the importance of considering disease activity and metabolic context when targeting this pathway, in order to avoid opposing biological outcomes under different inflammatory conditions.

## Supplementary Material

Supplementary figures and tables.

## Data Availability

The RNA sequencing data generated in this study have been deposited in the Gene Expression Omnibus (GEO) database under accession number GSE315761. Public datasets utilized in the analyses are also available from GEO. All other relevant raw data are available from the corresponding author upon reasonable request (yuanlianwen@csu.edu.cn).
