# Supplementary material for: A context-dependent METTL1-m7G-SLC7A11 axis links metabolic stress to epithelial fate in ulcerative colitis
Source: Int J Biol Sci. 2026 May 11;22(10):5142–60. doi: 10.7150/ijbs.133562 (PMC13215248; doi:10.7150/ijbs.133562)
Supplement: Supplementary file 1 — Supplementary figures and tables. [file ijbsv22p5142s1.pdf]

Supplementary Figure S1. METTL1/m^{7}G/SLC7A11 axis regulates metabolic reprogramming and oxidative stress in a glucose-dependent manner in intestinal epithelial cells. (A–C) Effects of METTL1 manipulation on cellular metabolic status under inflammatory conditions. Intestinal epithelial cells were treated with LPS under glucose-replete (LPS+Glc$^+$) or glucose-deprived (LPS+Glc$^-$) conditions. The GSH/GSSG ratio, NADP$^+$/NADPH ratio, and intracellular ATP levels were measured following METTL1 knockdown (sh-METTL1) or overexpression (OE-METTL1). (C) Rescue experiments showing that SLC7A11 knockdown attenuates METTL1 overexpression–induced metabolic alterations. (D–F) Effects of SLC7A11 manipulation on cellular metabolic status under the same conditions. Cells were subjected to SLC7A11 knockdown (sh-SLC7A11) or overexpression (OE-SLC7A11), followed by measurement of GSH/GSSG ratio, NADP$^+$/NADPH ratio, and ATP levels. (F) Treatment with the reducing agent TCEP partially rescues SLC7A11 overexpression–induced redox imbalance and energy depletion. (G, H) Intracellular reactive oxygen species (ROS) levels in FHC (G) and NCM460 (H) cells under different treatment conditions, detected using DCFH-DA fluorescent probe staining. Representative fluorescence images are shown. (I) Validation of disulfidptosis-associated cytoskeletal alterations in NCM460 cells under glucose deprivation and inflammatory stimulation, as indicated by F-actin staining. Data are presented as mean ± SD from three independent experiments. Statistical significance was determined by one-way ANOVA. *P < 0.05, **P < 0.01, ***P < 0.001, ****P < 0.0001; ns, not significant.

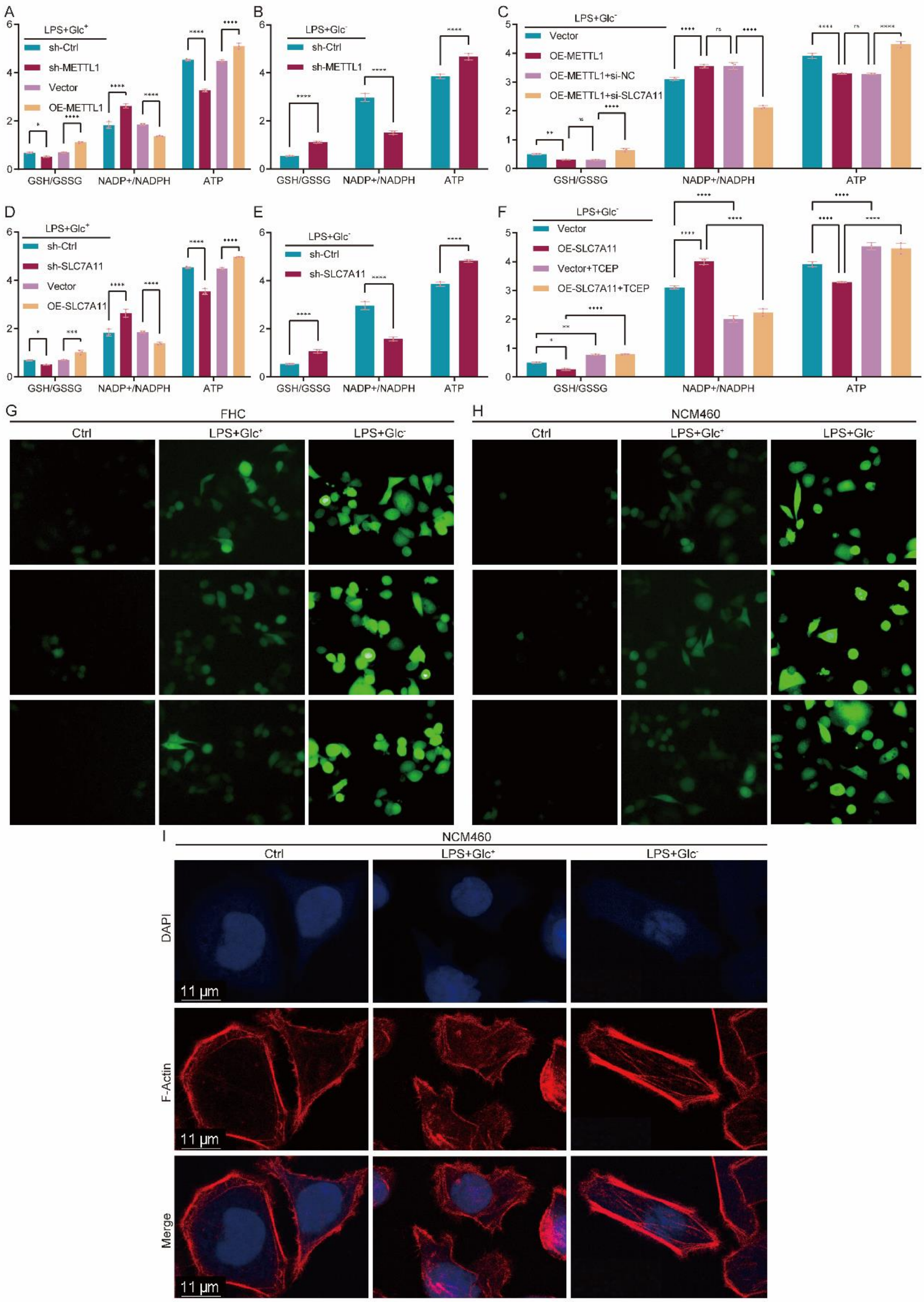

A
LPS+Glc+
sh-Ctrl
sh-METTL1
Vector
OE-METTL1
GSH/GSSG
NADP+/NADPH
ATP
B
LPS+Glc-
sh-Ctrl
sh-METTL1
GSH/GSSG
NADP+/NADPH
ATP
C
LPS+Glc-
Vector
OE-METTL1
OE-METTL1+si-NC
OE-METTL1+si-SLC7A11
GSH/GSSG
NADP+/NADPH
ATP
D
LPS+Glc+
sh-Ctrl
sh-SLC7A11
Vector
OE-SLC7A11
GSH/GSSG
NADP+/NADPH
ATP
E
LPS+Glc-
sh-Ctrl
sh-SLC7A11
GSH/GSSG
NADP+/NADPH
ATP
F
LPS+Glc-
Vector
OE-SLC7A11
Vector+TCEP
OE-SLC7A11+TCEP
GSH/GSSG
NADP+/NADPH
ATP
G
FHC
Ctrl
LPS+Glc+
LPS+Glc-
H
NCM460
Ctrl
LPS+Glc+
LPS+Glc-
I
NCM460
Ctrl
LPS+Glc+
LPS+Glc-
DAPI
F-Actin
Merge
11 µm

Antibody-data

WB/IF/IHC			
METTL1	Proteintech	84272-1-RR	
METTL1	Proteintech	14994-1-AP	
SLC7A11	Proteintech	26864-1-AP	
SLC7A11	Affinity	DF12509-50	
m7G	Proteintech	68302-1-Ig	
GLUT1	Proteintech	66290-1-Ig	
GPX4	Proteintech	67763-1-Ig	
GAPDH	Proteintech	60004-1-Ig	
α-Tubulin	Affinity	AF4651	
ELISA			
TNF-alpha	Proteintech	KE00154	Human
TNF-alpha	Proteintech	KE10002	Mouse
IL-6	Proteintech	KE00385	Human
IL-6	Proteintech	KE10007	Mouse
IL-1 beta	Proteintech	KE00021	Human
IL-1 beta	Proteintech	KE10003	Mouse
IL-18	Proteintech	KE00193	Human
IL-18	Invitrogen	A35613	Mouse

Adenoviral sequence

Gene	Target Seq
AAV9-sh-Ctrl	TTCTCCGAACGTGTCACGT
AAV9-sh-METTL1-1	GTTCTTTGCTCCGCTTATTCA
AAV9-sh-METTL1-2	CAAGTGGAGTTTGCAGACATA
AAV9-sh-METTL1-3	AGCTGCATGAGTGGATGTGCA
AAV9-sh-SLC7A11-1	AAGAAGTAGACAACCCTGAAA
AAV9-sh-SLC7A11-2	TGTTCGCTGTCTCCAGGTTAT
AAV9-sh-SLC7A11-3	AGATATGCATCGTCCTTTCAA

Lentiviral sequences(sh)

**Gene**	**Target Seq**
si-Ctrl	TTCTCCGAACGTGTCACGT
si-METTL1-1	GAATATGCCTACGTGCTAAGA
si-METTL1-2	GCAGACATAGGCTGTGGCTAT
si-METTL1-3	AGCTATACCCAGAGTTCTTCG
si-SLC7A11-1	CCTGTCACTATTTGGAGCTTT
si-SLC7A11-2	GCAGCTACTGCTGTGATATCC
si-SLC7A11-3	GCTCACAGCAATTCTGATAAT
**Vector name**	GV493
**Element order**	hU6-MCS-CBh-gcGFP-IRES-puromycin

Lentiviral sequences(OE)

Lentiviral overexpression vector	Gene	Forward primer	Reverse primer
pLenti-EF1a-C-Myc-DDK-IRES-Puro	METTL1	GCTCTAGAatggcagccgagactcggaa	GGAATTCcagtgaccaggcaggctggt
pLenti-EF1a-C-Myc-DDK-IRES-Puro	SLC7A11	GCTCTAGAatggtcagaaagcctgttgt	GGAATTCtcataacttatcttcttctg
CDS			

METTL1	atggcagc cgagactcgg aacgtggccg gagcagaggc<br>61 cccaccgccc cagaagcgct actaccggca acgtgctcac tccaacccca tggcggacca<br>121 cacgctgcgc taccctgtga agccagagga gatggactgg tctgagctat acccagagtt<br>181 cttcgctcca ctcactcaaa atcagagcca cgatgaccca aaggataaga aagaaaagag<br>241 agctcaggcc caagtggagt ttgcagacat aggctgtggc tatggtggcc tgttagtgga<br>301 actgtcaccg ctgttcccag acacacttat tctgggtctg gagatccggg tgaaggtctc<br>361 agactatgta caagaccgga ttcgggccct acgcgcagct cctgcaggtg gcttccagaa<br>421 catcgcctgt ctccgtagca atgccatgaa gcaccttcct aacttcttct acaagggcca<br>481 gctgacaaag atgttcttcc tcttccccga cccacatttc aagcggacaa agcacaagtg<br>541 gcgaatcatc agtcccaccc tgctagcaga atatgcctac gtgctaagag ttgggggggct<br>601 ggtgtatacc ataaccgatg tgctggagct acacgactgg atgtgcactc atttcgaaga<br>661 gcacccactg		

	tttgagcgtg tgcctctgga ggacctgagt gaagaccccg ttgtgggaca<br>721 tctaggcacc tcaactgagg aggggaagaa agttctacgt aatggaggga agaatttccc<br>781 agccatcttc cgaagaatac aagatcccgt cctccaggca gtgacctccc aaaccagcct<br>841 gcctggtcac tga		

SLC7A11	atggtcagaa agcctgttgt<br>301 gtccaccatc tccaaaggag gttacctgca gggaaatgtt aacgggaggc tgccttccct<br>361 gggcaacaag gagccacctg ggcaggagaa agtgcagctg aagaggaaag tcactttact<br>421 gaggggagtc tccattatca ttggcaccat cattggagca ggaatcttca tctctcctaa<br>481 gggcgtgctc cagaacacgg gcagcgtggg catgtctctg accatctgga cggtgtgtgg<br>541 ggtcctgtca ctatttggag ctttgtctta tgctgaattg ggaacaacta taaagaaatc<br>601 tggaggtcat tacacatata ttttggaagt ctttggtcca ttaccagctt ttgtacgagt<br>661 ctgggtggaa ctcctcataa tacgccctgc agctactgct gtgatatccc tggcatttgg<br>721 acgctacatt ctggaaccat tttttattca atgtgaaatc cctgaacttg cgatcaagct<br>781 cattacagct gtgggcataa ctgtagtgat ggtcctaaat agcatgagtg tcagctggag<br>841 cgcccggatc cagattttct taacctttttg caagctcaca gcaattctga taattatagt<br>901 ccctggagtt atgcagctaa ttaaaggtca		

	aacgcagaac tttaaagacg ccttttcagg<br>961 aagagattca agtattacgc ggttgccact ggctttttat tatggaatgt atgcatatgc<br>1021 tggctggttt tacctcaact ttgttactga agaagtagaa aaccctgaaa aaaccattcc<br>1081 ccttgcaata tgtatatcca tggccattgt caccattggc tatgtgctga caaatgtggc<br>1141 ctactttacg accattaatg ctgaggagct gctgctttca aatgcagtgg cagtgacctt<br>1201 ttctgagcgg ctactgggaa atttctcatt agcagttccg atctttgttg ccctctcctg<br>1261 ctttggctcc atgaacggtg gtgtgtttgc tgtctccagg ttattctatg ttgcgtctcg<br>1321 agagggtcac cttccagaaa tcctctccat gattcatgtc cgcaagcaca ctcctctacc<br>1381 agctgttatt gttttgcacc ctttgacaat gataatgctc ttctctggag acctcgacag<br>1441 tcttttgaat ttcctcagtt ttgccaggtg gctttttatt gggctggcag ttgctgggct<br>1501 gatttatctt cgatacaaat gcccagatat gcatcgtcct ttcaaggtgc cactgttcat<br>1561 cccagctttg ttttccttca catgcctctt		

	catggttgcc ctttccctct attcggaccc<br>1621 atttagtaca gggattggct tcgtcatcac tctgactgga gtccctgcgt attatctctt<br>1681 tattatatgg gacaagaaac ccaggtggtt tagaataatg tcggagaaaa taaccagaac<br>1741 attacaaata atactggaag ttgtaccaga agaagataag ttatga		

qPCR primer sequences

Qpcr	
mus-METTL1-F	GAACATCGCCTGTCTCCGAA
mus-METTL1-R	TCGCTTAAAGTGTGGGTCCG
mus-SLC7A11-F	GTTCGCTGTCTCCAGGTTATTCTAC
mus-SLC7A11-R	AGAGCATCACCATCGTCAGAGG
mmu-TNF-α F	CCCTCACACTCAGATCATCTTCT
mmu-TNF-α R	GCTACGACGTGGGCTACAG
mmu-IL-6 F	TAGTCCTTCCTACCCCAATTTCC
mmu-IL-6 R	TTGGTCCTTAGCCACTCCTTC
mmu-IL-18 F	GACTCTTGCGTCAACTTCAAGG
mmu-IL-18 R	CAGGCTGTCTTTTGTCAACGA
mmu-IL-1β F	GCAACTGTTCCTGAACTCAACT
mmu-IL-1β R	ATCTTTTGGGGTCCGTCAACT
mmu-GAPDH F	AGGTCGGTGTGAACGGATTTG
mmu-GAPDH R	TGTAGACCATGTAGTTGAGGTCA
hs-METTL1-F	CCGACCCACATTTCAAGCG
hs-METTL1-R	TCCAGCACATCGGTTATGGTA
hs-SLC7A11-F	TTTGTTGCCCTCTCCTGCTTTG
hs-SLC7A11-R	AGTGTGCTTGCGGACATGAATC
hs-GPX4-F	CAGTGAGGCAAGACCGAAGT
hs-GPX4-R	CCGAACTGGTTACACGGGAA
hs-TNF-α-F	TCAGCAAGGACAGCAGAGGAC
hs-TNF-α-R	GGTGGAGCCGTGGGTCAG
hs-IL-6-F	TTCGGTCCAGTTGCCTTCTCC
hs-IL-6-R	TTCTGAAGAGGTGAGTGGCTGTC
hs-IL-18-F	CCTGGACAGTCAGCAAGGAATTG
hs-IL-18-R	AGGAAGCGATCTGGAAGGTCTG
hs-IL-1β-F	TGGCTTATTACAGTGGCAATGAGG
hs-IL-1β-R	AGTGGTGGTCGGAGATTCGTAG
hs-GAPDH-F	CAGGAGGCATTGCTGATGAT
hs-GAPDH-R	GAAGGCTGGGGCTCATTT

m7G MeRIP Qpcr-SLC7A11	
hs-SLC7A11-1-F	AACCTTTTGCAAGCTCACAGC
hs-SLC7A11-1-R	GTGGCAACCGCGTAATACTT
hs-SLC7A11-2-F	CGCCCGGATCCAGATTTTCTT
hs-SLC7A11-2-R	GTGGCAACCGCGTAATACTTG
hs-SLC7A11-3-F	AGTCCCTGGAGTTATGCAGC
hs-SLC7A11-3-R	TGGCAACCGCGTAATACTTG
